# Midterm postoperative outcomes of different types of surgical reconstruction of sinus venosus atrial septal defects with anomalous pulmonary venous connection: The Results of Prospective Cohort Study

**DOI:** 10.1002/hsr2.990

**Published:** 2022-12-22

**Authors:** Vishal V. Bhende, Tanishq S. Sharma, Deepakkumar V. Mehta, Bhadra Y. Trivedi, Amit Kumar, Viral B. Patel, Gurpreet Panesar, Kunal Soni, Kartik B. Dhami, Nirja Patel, Sohilkhan R. Pathan, Hardil P. Majmudar

**Affiliations:** ^1^ Department of Pediatric Cardiac Surgery, Bhanubhai and Madhuben Patel Cardiac Centre, Shree Krishna Hospital Bhaikaka University Anand Gujarat India; ^2^ Department of Radiodiagnosis & Imaging, Pramukhswami Medical College & Shree Krishna Hospital Bhaikaka University Anand Gujarat India; ^3^ Department of Pediatric Cardiology, Bhanubhai and Madhuben Patel Cardiac Centre, Shree Krishna Hospital Bhaikaka University Anand Gujarat India; ^4^ Department of Pediatric Cardiac Intensive Care, Bhanubhai and Madhuben Patel Cardiac Centre, Shree Krishna Hospital Bhaikaka University Anand Gujarat India; ^5^ Department of Cardiac Anaesthesiology, Bhanubhai and Madhuben Patel Cardiac Centre, Shree Krishna Hospital Bhaikaka University Anand Gujarat India; ^6^ Department of Clinical Research Services, Bhanubhai and Madhuben Patel Cardiac Centre, Shree Krishna Hospital Bhaikaka University Anand Gujarat India

**Keywords:** anomalous pulmonary venous connection, complications, single‐patch technique, sinus node block, sinus venosus atrial septal defects, two‐patch technique

## Abstract

**Background and Aims:**

Sinus venosus atrial septal defects (SVASDs) constitute a substantial part of atrial septal defects and are usually characterized by anomalous pulmonary venous connection (APVC), causing complications like sinus node dysfunction and arrhythmias. Several surgical approaches are used for treating SVASDs in pediatric patients, including single‐ and two‐patch techniques. The study aimed to prospectively evaluate and compare the safety and efficacy of these two methods with different follow‐up periods.

**Methods:**

Ten patients aged 1–8 years with SVASDs and partial APVC were enrolled in the study at Bhanubhai and Madhuben Patel Cardiac Centre, Karamsad, India, between December 2018 and October 2021. The single‐patch (sandwich‐patch) technique was used in two patients, whereas the two‐patch (dual‐patch) technique with autologous pericardium was used in seven. Safety was assessed as the absence of complications in the follow‐up periods of 6 months, 1, and 2 years, whereas efficacy was estimated by the preserved sinus rhythm and the development of arrhythmias. Electrocardiographic and echocardiographic methods were used to evaluate both parameters.

**Results:**

No deaths, reoperations, pulmonary vein, and superior vena cava (SVC) stenosis or phrenic nerve palsy were observed among the 10 patients in the three follow‐up periods. Sinus rhythm was arrested in two of the seven patients who underwent two‐patch repair, whereas no rhythm disturbances occurred in those who underwent single‐patch repair.

**Conclusion:**

Both techniques used in SVASD repair with autologous pericardium proved to cause the smaller rate of complications in midterm postsurgical phase. However, there is a potentially great risk of the development of sinus node malfunction after the application of the two‐patch technique. Therefore, methods avoiding sinus node interference are preferred in patients with partial APVC involving SVC.

## 1 INTRODUCTION

Sinus venosus atrial septal defects (SVASDs) are relatively rare congenital heart defects belonging to one of the major groups of atrial septal defects (ASDs). They were first described in 1858 and compose around 4%–11% of ASD cases.^1^, ^2^ SVASD can be described as the deficiency of connective tissue at the posterior aspect of the atrial septum that is clinically manifested as atrial communication with the superior or inferior vena cava (IVC). In the former case, they are usually accompanied by anomalous pulmonary venous connections (APVC) from the right superior pulmonary vein (RSPV) to the SVC.^3^, ^4^ Left‐to‐right shunt is produced because the left atrial pressure exceeds that of the right, and long‐term effects are associated with the overload of the right heart, such as pulmonary hypertension.

The major surgical principle of treatment is providing the bypass for the venous flow to the left atrium (LA) by redirecting APVC through the junction of the left and right atriums (RAs).

Surgical interventions possess certain risks due to the complexity of the performed procedures compared to surgical reconstruction of secundum ASD. The major complications in the postoperative period include the risk of reduced blood flow in the SVC or pulmonary veins, residual shunting, and sinus node malfunction.^4^

**Figure 1:**
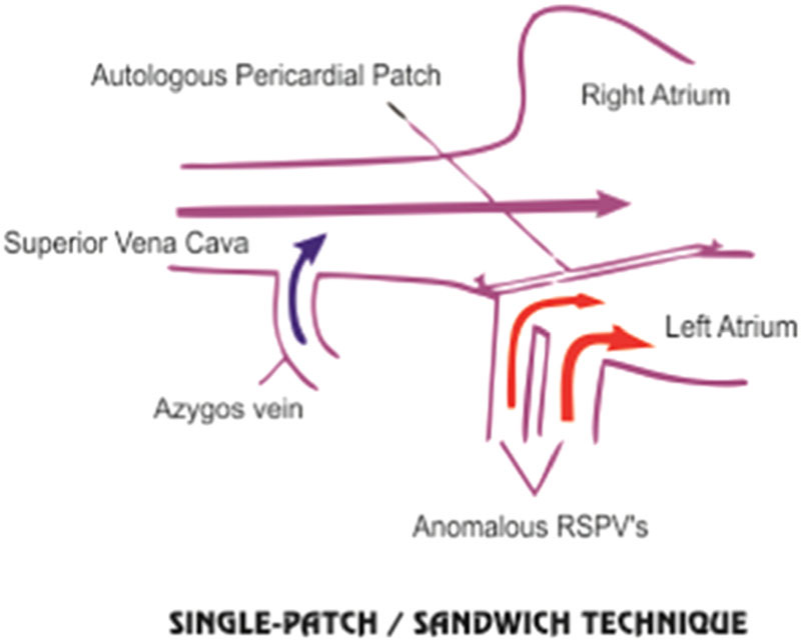
Single‐patch/sandwich‐patch technique. RSPV, right superior pulmonary vein (Image credits: Dr. Vishal V. Bhende).

**Figure 2:**
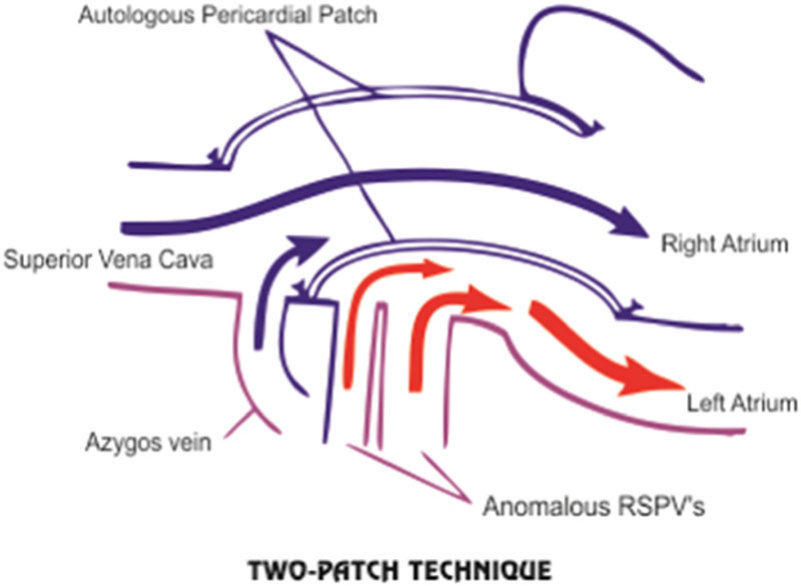
Two‐patch (dual‐patch) technique. RSPV, right superior pulmonary vein (Image credits: Dr. Vishal V. Bhende).

The two common surgical methods used for the treatment of malformation are single‐ and two‐patch techniques (Figures 1 and 2).

Due to the abnormal direct connection of pulmonary veins with SVC, the latter is the more common method of choice. In this technique, one patch is used to close the ASD and another to close the right atriotomy at the cavoatrial junction to decrease the risk of stenosis.^5^

Although most reported studies are in favor of the better efficacy of the former, there is no unified consensus on the application of these methods in particular clinical cases. Most existing works focus on the long‐term prognosis of their application, whereas the complications in the earlier postoperative time can indicate the safety of the technique. Therefore, this study aimed to assess the rate of complications and sinus dysfunction in midterm follow‐up periods as the determinant factors for choice in clinical situations.

## 2 METHODS

Ten patients from our computerized cardiovascular database (FTP 192.168.0.5) who underwent primary surgical repair of SVASD at Bhanubhai and Madhuben Patel Cardiac Centre, Karamsad, Anand, Gujarat, India, between December 2018 and October 2021 were included.

The Institutional Ethics Committee (IEC‐2) of the Bhanubhai and Madhuben Patel Centre for Medical Care and Education, Anand, Gujarat, India, gave the approval for this study [No. (IEC/BU/2021/Cr.48/283) dated 19.11.2021].

**Figure 3:**
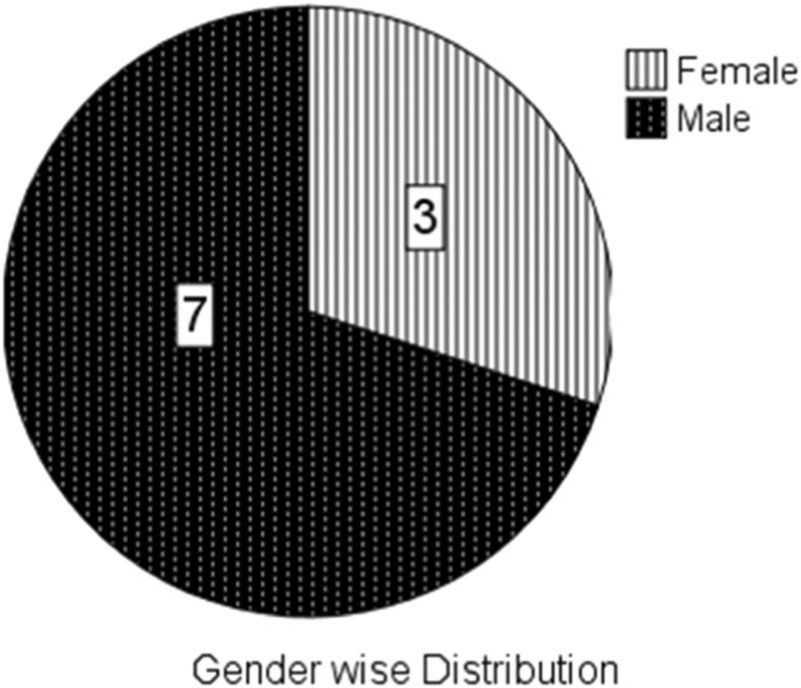
Gender distribution of patients in this study

**Figure 4:**
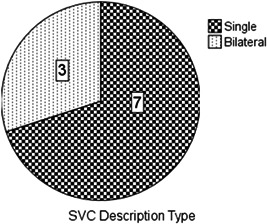
Type of superior vena cava (SVC)

The mean age of patients was 3.7 years (median, 3.5; range, 1–8 years). Seven were boys and three were girls. The gender distribution of patients and type of superior vena cava (SVC) are illustrated in (Figures 3 and 4).

One patient (P10) had left upper partial anomalous pulmonary venous connection (PAPVC) without SVASD and was included in the study for comparison. In nine patients with SVASDs, the PAPVC drainage was through the RA‐SVC communication; in patient P10, partial anomalous venous drainage through a vertical vein was exhibited at the left upper lobe of the lung, which in turn drained into the brachiocephalic vein (Table 1).

**Table 1:** Patients characteristics undergoing repair of sinus venosus atrial septal defect through various techniques

Sr. No.	Patient	Age at time of repair (years)	Sex	Weight (kg)	SVC	CPB time (min)	ACC time (min)	Technique	Length of hospitalization after DOSx (days)	Intraoperative pressures	2D echocardiographic findings at discharge	Complications
1	P1	4	Female	11.8	Single	104	71	Single‐patch	5	SVC: 16/13 (15) mmHg SVC‐RA junction: 15 mmHg Mid‐RA cavity: 11/04 (08) mmHg	Flow acceleration at SVC‐RA junction with 4 mmHg of peak gradient and 2 mmHg of mean gradient	—
2	P2	1	Male	6.9	Single	98	68	Single‐patch	8	—	Turbulence at SVC‐RA junction with 18 mmHg of peak and mean gradient	—
3	P3	1	Male	7.8	Bilateral	98	68	Two‐patch	8	SVC: 9 mmHg RA: 2 mmHg	Turbulence at SVC‐RA junction with 8.5 mmHg of peak gradient and 4.2 mmHg of mean gradient	—
4	P4	1	Female	6.8	Single	97	66	Two‐patch	10	SVC: 5 mmHg RA: 1 mmHg	Turbulence from SVC to RA with 9 mmHg of peak gradient and 5 mmHg of mean gradient	—
5	P5	8	Male	18.40	Single	120	77	Two‐patch	10	SVC: 5 mmHg RA: 2 mmHg	WNL	—
6	P6	3	Male	12.0	Single	103	79	Two‐patch	8	SVC: 8 mmHg RA: 8 mmHg	WNL	—
7	P7	4	Female	12.0	Bilateral	113	82	Two‐patch	9	SVC: 8 mmHg RA: 5 mmHg	WNL	—
8	P8	5	Male	15.39	Single	84	54	Two‐patch	8	SVC: 5 mmHg RA: 10 mmHg	WNL	—
9	P9	3	Male	11.4	Bilateral	117	67	Two‐patch	8	SVC: 9 mmHg RA: 5 mmHg	WNL	—
10	P10	7	Male	20.0	Single	150	106	Left PAPVC rerouting, ASD closure, and pulmonary valvotomy	6	—	WNL	—

Abbreviations: 2D, two‐dimensional; ACC, aortic cross‐clamping; ASD, atrial septal defect; CPB, cardiopulmonary bypass; DOSx, date of surgery; PAPVC, partial anomalous pulmonary venous connection; RA, right atrium; SVC, superior vena cava; WNL, within normal limits.

The cases where severe pulmonary hypertension and other contraindications for surgery were observed, along with the right upper pulmonary vein (RUPV) being from the cavo‐atrial junction at the distance >1 cm, were excluded from the study. Patients aged <1 year did not participate in the study. No arrhythmias were present in the presurgical period in patients.

### 2.1 Preoperative phase

**Figure 5:**
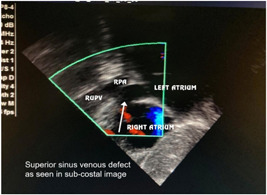
Preoperative two‐dimensional echocardiogram for patient P9. RPA, right pulmonary artery; RUPV, right upper pulmonary vein (Image credits: Dr. Bhadra Y. Trivedi).

**Figure 6:**
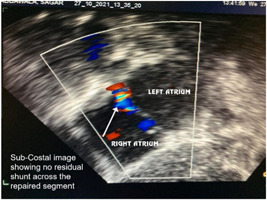
Postoperative two‐dimensional echocardiogram for patient P9 (Image credits: Dr. Bhadra Y. Trivedi).

**Figure 7:**
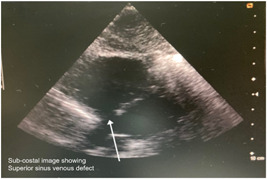
Preoperative two‐dimensional echocardiogram for patient P1 (Image credits: Dr. Bhadra Y. Trivedi).

**Figure 8:**
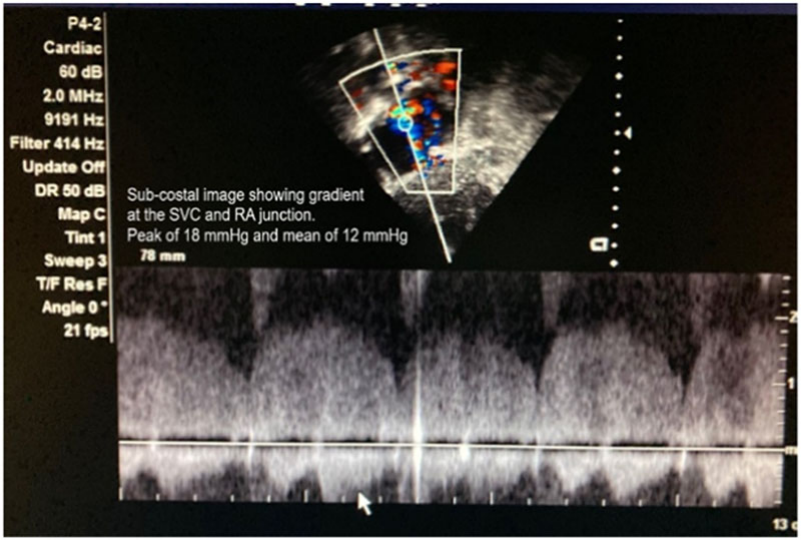
Postoperative two‐dimensional echocardiogram for patient P2. RA, right atrium; SVC, superior vena cava (Image credits: Dr. Bhadra Y. Trivedi).

In the preoperative period, all 10 patients enrolled in the study underwent two‐dimensional (2D) echocardiography (Figures 5, 6, 7, 8).

In all patients, preoperative electrocardiography (ECG) was also performed.

### 2.2 Dynamic cardiac computed tomography (CT)

**Figure 9:**
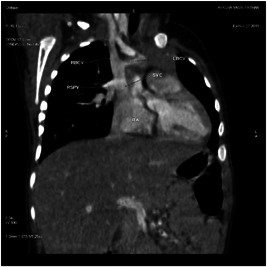
Maximum‐intensity projection, coronal view, in dynamic cardiac computed tomography of the RSPV opening in the SVC in patient P4. LBCV, left brachiocephalic vein; RA, right atrium; RBCV, right brachiocephalic vein; RSPV, right superior pulmonary vein; SVC, superior vena cava (Image credits: Dr. Deepakkumar V. Mehta).

**Figure 10:**
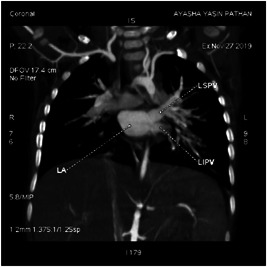
Maximum‐intensity projection, coronal view, in dynamic cardiac computed tomography of LA, LSPV, and LIPV in patient P4. LA, left atrium; LIPV, left inferior pulmonary vein; LSPV, left superior pulmonary vein (Image credits: Dr. Deepakkumar V. Mehta).

**Figure 11:**
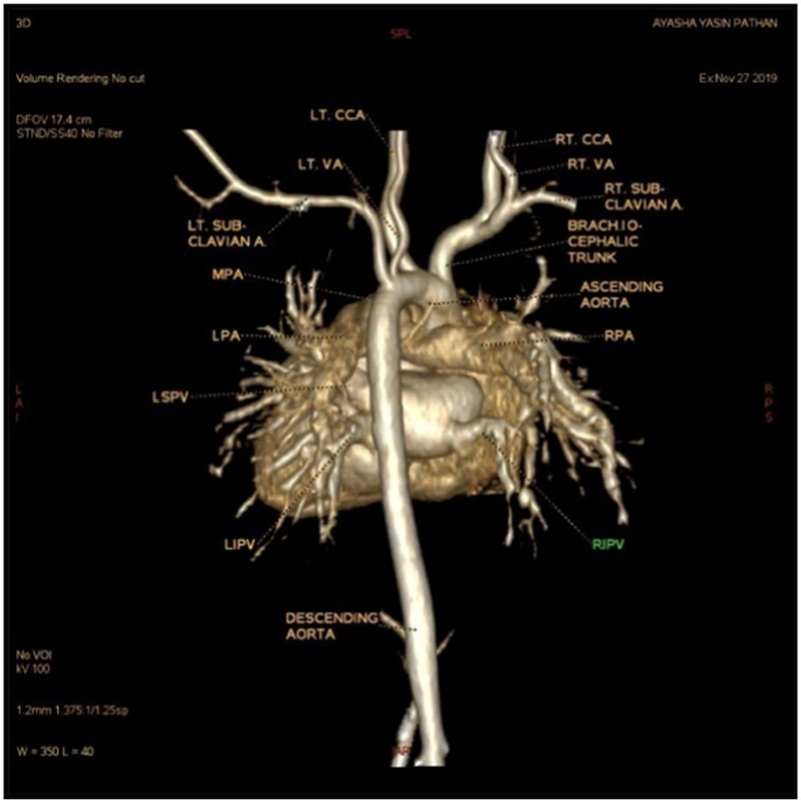
Volume rendering, anterior view, in dynamic cardiac computed tomography patient P4. CCA, common carotid artery; LIPV, left inferior pulmonary vein; LPA, left pulmonary artery; LSPV, left superior pulmonary vein; Lt., left; MPA, main pulmonary artery; RIPV, right inferior pulmonary vein; Rt., right; RVA, right vertebral artery (Image credits: Dr. Deepakkumar V. Mehta).

**Figure 12:**
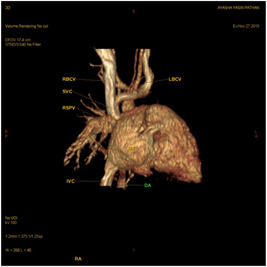
Volume rendering, posterior view, in dynamic cardiac computed tomography in patient P4. DA, ductus arteriosus; IVC, inferior vena cava; LBCV, left brachiocephalic vein; RBCV, right brachiocephalic vein; RSPV, right superior pulmonary vein; SVC, superior vena cava (Image credits: Dr. Deepakkumar V. Mehta).

Dynamic cardiac CT was performed preoperatively in our series for two patients. In patient P4, intracranial arteriovenous malformations were strongly suspected of leading to SVC dilatation. Dynamic cardiac CT revealed a moderately large ASD (superior sinus venosus type) (Figures 9, 10, 11, 12).

The RSPV drained into the terminal portion of the SVC. This scenario was suggestive of both PAPVC and PAPVD. The main, right, and left pulmonary arteries were dilated, which was suggestive of pulmonary arterial hypertension. The left vertebral artery (LVA) ascended from the aortic arch as a third branch. The proximal, middle, and distal diameters of the SVC each measured approximately 8.7 mm.

**Figure 13:**
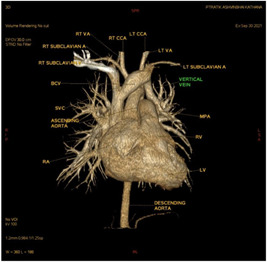
Volume rendering, anterior view, in dynamic cardiac computed tomography in patient P10. BCV, brachiocephalic vein; CCA, common carotid artery; Lt., left; LV, left ventricle; MPA, main pulmonary artery; RA, right atrium; Rt., right; RV, right ventricle; SVC, superior vena cava; VA, vertebral artery (Image credits: Dr. Viral B. Patel).

**Figure 14:**
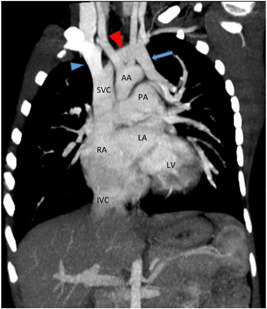
Maximum‐intensity projection, coronal view, in pulmonary angiography in patient P10. Vertical vein (blue arrow; PAPVC from left upper lobe of lung) drained into the left brachiocephalic vein (red arrowhead). The blue arrowhead signifies the right brachiocephalic vein. AA, arch of aorta; IVC, inferior vena cava; LA, left atrium; LV, left ventricle; PA, pulmonary artery; PAPVC, partial anomalous pulmonary venous connection; RA, right atrium; SVC, superior vena cava (Image credits: Dr. Viral B. Patel).

In patient P10, left‐sided partial pulmonary venous connection to the innominate vein was suspected, and cardiac CT and pulmonary angiography were performed to confirm this (Figures 13 and 14).

Dynamic cardiac CT of the upper lobe of the left lung revealed partial anomalous venous drainage through a vertical vein, which in turn drained into the brachiocephalic vein; this scenario was suggestive of PAPVD. The hemiazygos vein drained posteriorly into an anomalous vertical vein at the lower border of the T4 vertebral level, which was 1.7 cm below the confluence of the anomalous vertical vein and the brachiocephalic vein. The distance of 1.3 cm was there from the anomalous vein hilum to the opening of the hemiazygos vein. The flux from other pulmonary veins was directed to the LA.

### 2.3 Surgical phase

#### 2.3.1 Surgical techniques

Median sternotomy was performed in all the cases. In seven patients, bicaval cannulation was applied, and in three patients, tricaval cannulation was used for cardiopulmonary bypass with ascending aortic cannulation, whereas moderate systemic hypothermia (25–32°C) was used in all patients.

**Figure 15:**
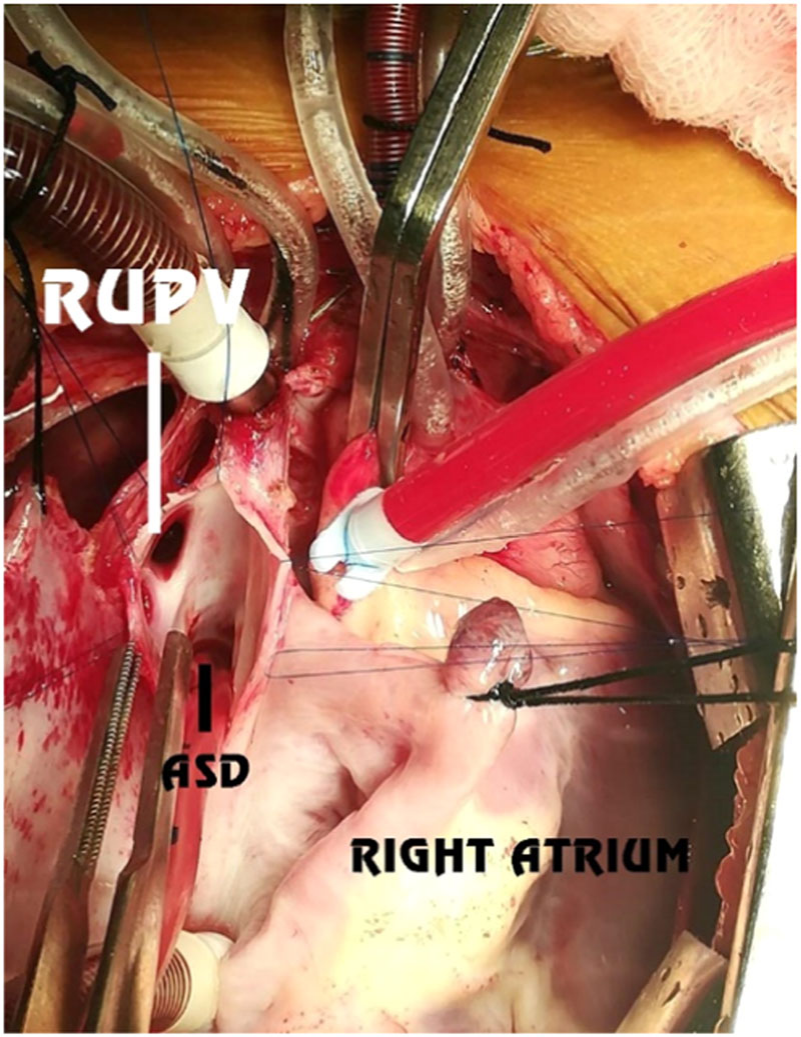
Sinus venosus atrial septal defect in patient P1. ASD, atrial septal defect; RUPV, right upper pulmonary vein (Image credits: Dr. Vishal V. Bhende).

**Figure 16:**
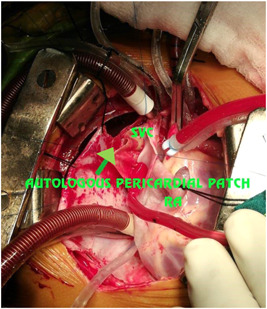
Single‐patch for patient P1. RA, right atrium; SVC, superior vena cava (Image credits: Dr. Vishal V. Bhende).

**Figure 17:**
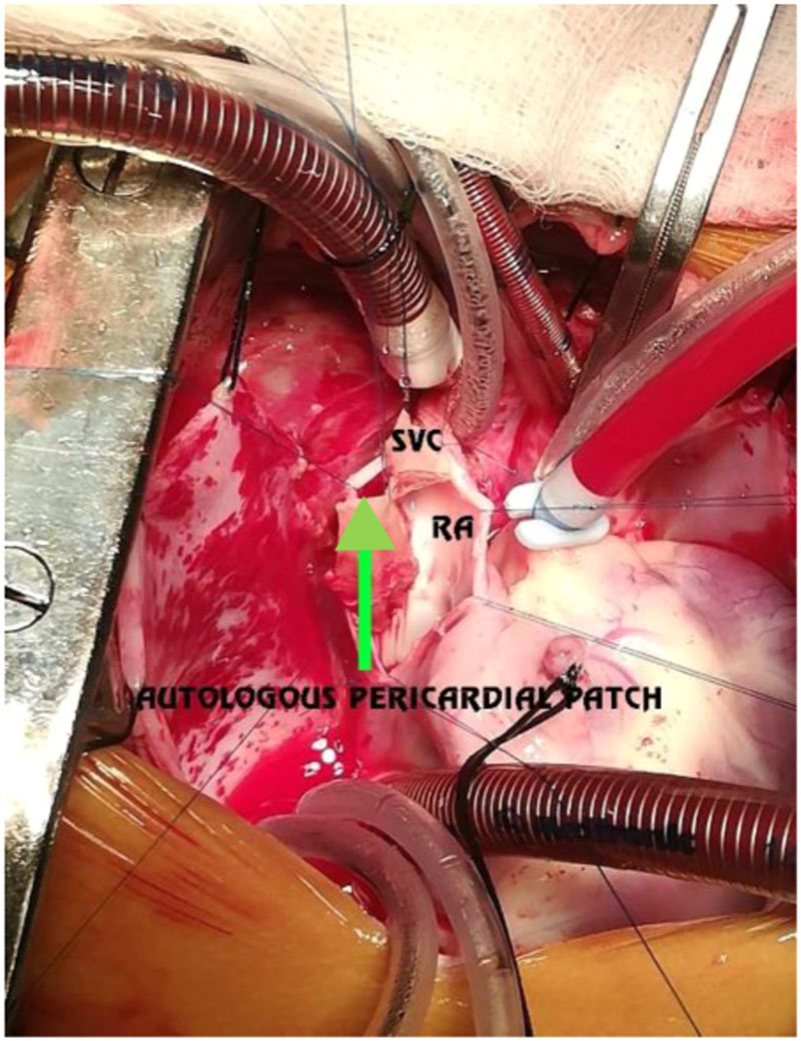
Single‐patch for patient P2. RA, right atrium; SVC, superior vena cava (Image credits: Dr. Vishal V. Bhende).

**Figure 18:**
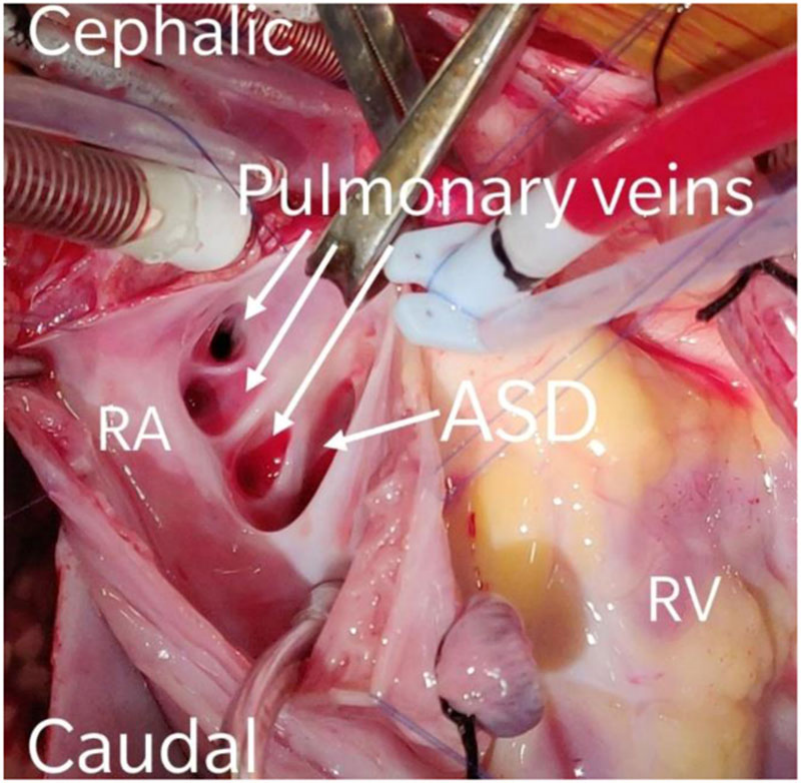
SVASD in patient P7. ASD, atrial septal defect; RA, right atrium; RV, right ventricle; SVASD, sinus venosus atrial septal defects (Image credits: Dr. Vishal V. Bhende).

**Figure 19:**
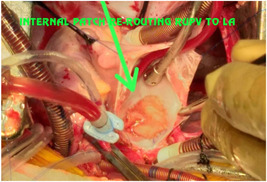
Two‐patch technique (internal patch) for patient P9. LA, left atrium; RUPV, right upper pulmonary vein (Image credits: Dr. Vishal V. Bhende).

**Figure 20:**
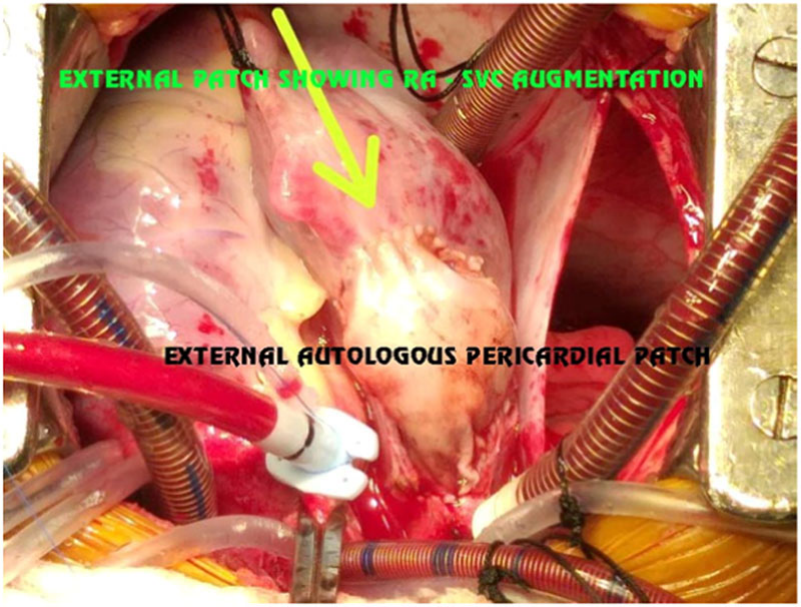
Two‐patch (external patch) technique for patient P9. RA, right atrium; SVC, superior vena cava (Image credits: Dr. Vishal V. Bhende).

**Figure 21:**
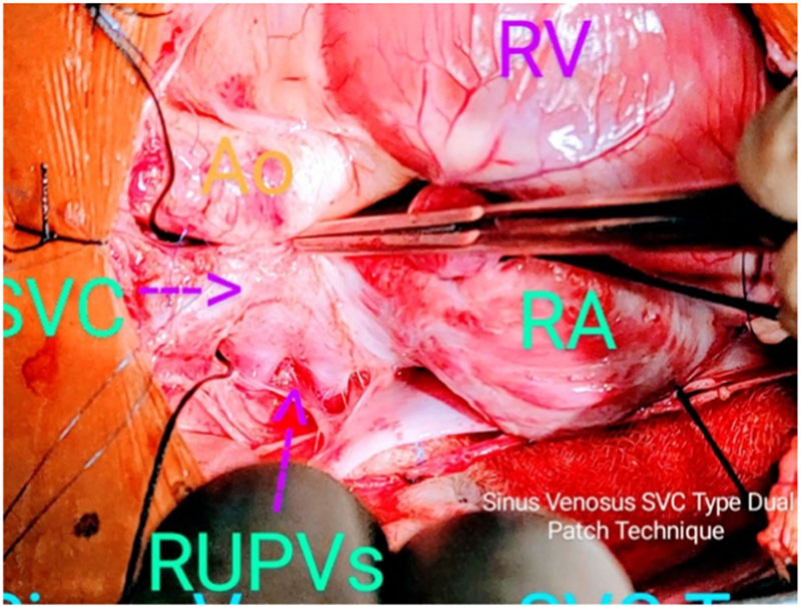
Sinus venosus atrial septal defect with RUPVs in patient P6. RA, RV, SVC are signified with arrows. RA, right atrium; RV, right ventricle; SVC, superior vena cava (Image credits: Dr. Vishal V. Bhende).

We achieved cardioplegic arrest with Del Nido cardioplegia and topical slush saline. We did not routinely use a left ventricular vent. All patches were made from the fresh autologous pericardium. In two patients (20%), we performed single‐patch repair of the SVASD with the inclusion of the PAPVC and ASD closure; in seven (70%), we performed repair with two pericardial patches to enlarge the SVC‐RA junction (Figures 15, 16, 17, 18, 19, 20, 21).

**Figure 22:**
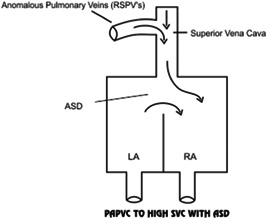
PAPVC is shown to a high point on the SVC with ASD. ASD, atrial septal defect; LA, left atrium; PAPVC, partial anomalous pulmonary venous connection; RA, right atrium; RSPV, right superior pulmonary vein (Image credits: Dr. Vishal V. Bhende).

**Figure 23:**
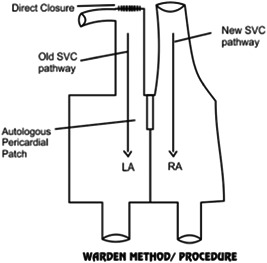
Warden procedure. LA, left atrium; RA, right atrium; SVC, superior vena cava (Image credits: Dr. Vishal V. Bhende).

**Figure 24:**
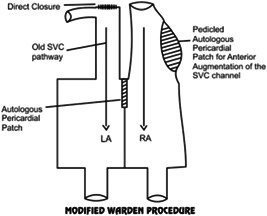
Modified Warden procedure. ASD, atrial septal defect; LA, left atrium; RA, right atrium; SVC, superior vena cava (Image credits: Dr. Vishal V. Bhende).

None of the patients in our series required the Warden procedure (Figures 22, 23, 24).

In patient P10, we rerouted the PAPVC by creating an anastomosis between the left atrial appendage and the vertical vein with flush ligation of the vertical vein and the innominate vein, leaving the hemiazygos vein in the systemic pathway. We used a glutaraldehyde‐treated pericardial patch to close the ASD and performed pulmonary valvotomy.

The intraoperative characteristics are represented in Table 1, including cardiopulmonary bypass and aortic cross‐clamping times.

### 2.4 Postsurgical phase

Complete follow‐up was obtained for all 10 patients at 6‐month, annual, and bi‐annual intervals.

#### 2.4.1 ECG findings

Initial postoperative ECG was regarded as early, while ECG after hospital discharge was denoted as late.

Two unaware of the patients' type of surgical repair reviewers interpreted patients' ECG patterns as normal sinus, low atrial, or junctional rhythms. Criteria for interpretation for low atrial rhythm were fulfilled by the redirected P‐wave axis or substantially decreased PR interval.

Persistent sinus bradycardia (<50 bpm), sinus rhythm disturbances, or a wandering atrial pacemaker (<60 bpm); pauses of >3 s were defined as sinus node dysfunction. Electrophysiological evidence was also used to diagnose sinus node dysfunction. New sinus node dysfunction was defined as the disturbance appearing postoperatively (present at hospital discharge or at the late postsurgical phase in the cases in which sinus rhythm had been present preoperatively).

#### 2.4.2 Echocardiographic findings

The 2D echocardiograms were evaluated at the time of discharge and at follow‐ups. Changes appearing at the SVC‐RA junction, such as flow acceleration, and changes in the peak gradient were assessed (Table 1).

### 2.5 PV and SVC stenosis assessment

The grading for the stenosis of SVC and RSPV was determined as trivial, mild, moderate, or severe. This parameter was assessed in the three follow‐up periods.

### 2.6 Statistical analysis

The data analysis was performed by STATA, version 14.2 (StataCorp. 2015. Stata Statistical Software: Release 14. College Station, TX, USA: StataCorp. LP).

The comparative analysis was done by Fisher's exact test using descriptive statistics to show an association between technique (single‐vs. two‐patch) and rhythm.

## 3 RESULTS

In this series performed between 2018 and 2021, no mortality was observed in the early or late postsurgical periods. Further, no residual ASDs were detected; therefore, no reoperations were required. Of the nine patients with PAPVC involving the RA‐SVC junction or RA, none had the restriction of blood flow in either the SVC or pulmonary veins on follow‐up echocardiography, and none required the Warden procedure. In our series, all the restorations with pericardium patches were successful.

**Figure 25:**
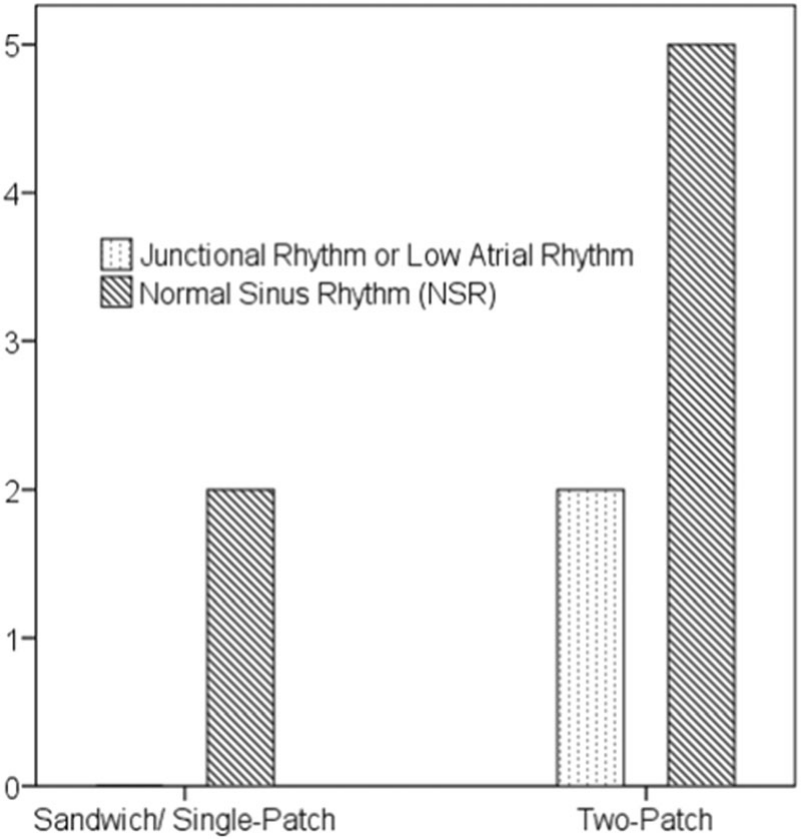
Comparison of postoperative and late electrocardiograms showing the change from normal sinus rhythm to low sinus node dysfunction

The association between technique (single‐ vs. two‐patch) and rhythm was not significant (*p* > 0.999; Figure 25).

**Figure 26:**
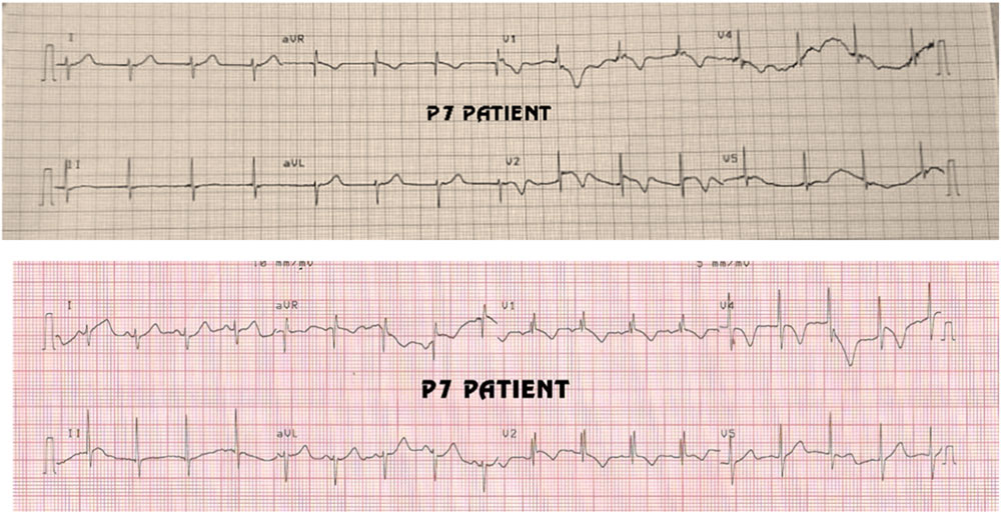
Electrocardiographic strips for patient P7. Top, junctional rhythm; bottom, normal sinus rhythm

Two patients, P4 and P7, who underwent two‐patch repair exhibited junctional rhythm on early ECG (Figure 26).

This junctional rhythm returned to sinus rhythm on late ECG in the hospital. The remaining eight patients displayed normal sinus rhythm on early ECG. The 2D echocardiographic findings of patient P2 on discharge revealed turbulence at the SVC‐RA junction with peak and mean gradients of 18 mmHg each. Subsequent echocardiographic findings of the same patient at the 6‐month, annual, and bi‐annual follow‐ups revealed diminishing gradients with peak and mean gradients of 10/6 mmHg. each. Patient P2 has been monitored for the last 3 years and continues to be free of SVC occlusion symptoms.

All seven patients who underwent two‐patch repair continue to be monitored at our cardiac center. In the two patients who underwent single‐patch repair, turbulence was noted in the SVC‐RA junction pathway: patient P1 exhibited peak and mean pressures of 4 and 2 mmHg, respectively, and P2 exhibited a peak and mean pressures of 18 mmHg each. No rhythm abnormalities occurred after single‐patch repair.^6^, ^7^

## 4 DISCUSSION

Since the earliest reports of the anomaly, the repair of SVASD with PAPVC involving the SVC was a serious issue imposing potentially high risk of complications.^8^, ^9^ To redirect pulmonary venous return, there are numerous surgical modifications. As reported in many surgical series, the major complications at the early stage have been mostly resolved, but the stenosis of SVC and pulmonary veins, along with sinus node dysfunction remains the same. In our study, PAPVC involving SVC at a low level, RA, or both was treated by septal transposition^10^ or by providing the bypass for the anomalous pulmonary venous flow to the LA through the ASD and with a patch applied from within the RA.^11^ We did not encounter cases in which PAPVC was involved with the SVC at a high level (≥1 cm above the cavoatrial junction) with or without the associated ASD.

In the literature review, we found a very low incidence of stenosis of the SVC or pulmonary veins with the use of the two‐patch technique. According to previous studies, there is a significant incidence of sinus node malfunction after two‐patch repair, which involves an incision crossing the SVC‐RA intercommunication anteriorly or laterally, thereby making potential damage for the sinus node. In a review by Delon et al.,^11^ sinus node dysfunction was more likely to occur in the group of patients who underwent repair with the technique implying the incisive creation of RA appendage flap at the anterior RA‐SVC junction.

Simliarly, the study by Stewart et al.^10^ showed that in the group of 15 patients with SVASD and PAPVC involving the SVC and undergoing surgical repair with a single‐patch method, only 6 of them developed postsurgical sinus node dysfunction that was eliminated before hospital discharge. The same finding is associated with one of our patients (P9), in whom normal sinus rhythm was reverted after the initial dysfunction.

In our study, patients who underwent two‐patch repair continued to exhibit normal sinus rhythm ECG in the late postoperative phase with no turbulence in the SVC‐RA junction pathway and no arrhythmias; therefore, we advocate the use of two‐patch repair over single‐patch repair.

According to our review of the literature, in the Warden procedure that requires no incision near the sinus node or sinus nodal artery, no sinus node dysfunction occurs after the procedure. Gustafson et al. and Warden et al. reported this in their study, where^12^, ^13^ the Warden procedure was performed in 40 patients, of whom only 1 had continuing sinus node dysfunction.^12^, ^13^ In contrast, only two of them had sinus node dysfunction in the early period, while the normal sinus rhythm was achieved by 5 days and 6 months after surgery.

In the pediatric cases of SVASD with PAPVC involving high levels of the SVC, the Warden procedure encourages the flow of blood in the SVC and pulmonary veins, possibly diminishing the risk of sinus node dysfunction that is associated with two‐patch repair; therefore, this procedure should be regarded as an option.

Even in patients aged >40 years, this procedure has lower risks, although it is characterized by multiple challenges. Although early repair is recommended, the survival rates say in favor for the procedure.^6^

A study by Shahriari et al.^14^ published the results of SVASD repair in 54 patients, of whom 27, 12 and 13 underwent single‐, two‐patch repair the Warden procedure, respectively. The arrhythmias occurred with very low frequency; all patients who had the Warden procedure had normal sinus rhythm. Additionally, in the series reported by Gaynor et al.,^15^ 11 patients who were treated with the Warden procedure between 1987 and 1995 did not exhibit sinus node dysfunction. A single case of transient sinus bradycardia among the group of patients who had the Warden procedure between 1995 and 2004 was reported by DiBardino and associates.^16^, ^17^ Thus, in the literature, it is clearly understood that sinus node injury and sinus node dysfunction can be eliminated through the Warden procedure.

In the above‐mentioned combined series, no patient had SVC stenosis, and only two had pulmonary vein stenosis. Notably, in both patients who were treated at different hospitals, pulmonary vein stenosis appeared, and polytetrafluoroethylene intra‐atrial baffle was recommended by the authors due to untreated shrinkage of the autologous pericardial patch.^14^, ^15^, ^16^

## 5 LIMITATIONS

The main limitation is connected with the small sample size and the study being performed at a single institution.

The frequency of sinus node dysfunction detection may have been underestimated, because atrial arrhythmias occur potentially decreasing the ability to detect sinus node dysfunction.

## 6 CONCLUSIONS

The decision to surgically repair SVASD should be individualized. Our findings confirm that for patients with SVASD and PAPVC involving the RA or the SVC‐RA junction, the two‐patch method remains preferrable, despite the higher risk of the development of sinus node dysfunction. In the cases of bilateral SVC without innominate vein connection, single‐patch repair can be attempted without causing much turbulence in the SVC‐RA junction in comparison with cases of single SVC.

## AUTHOR CONTRIBUTIONS

**Vishal V. Bhende**: Conceptualization; data curation; formal analysis; funding acquisition; investigation; methodology; project administration; resources; software; supervision; validation; visualization; writing – original draft; writing – review and editing. **Tanishq S. Sharma**: Conceptualization; data curation; formal analysis; funding acquisition; investigation; methodology; project administration; resources; software; supervision; validation; visualization; writing – original draft; writing – review and editing. **Deepakkumar V. Mehta**: Data curation; formal analysis; investigation; methodology; resources; software; supervision; validation; visualization. **Bhadra Y. Trivedi**: Data curation; formal analysis; investigation; methodology; project administration; resources; software; supervision; validation; visualization. **Amit Kumar**: Data curation; formal analysis; investigation; methodology; project administration; software; supervision; validation; visualization. **Viral B. Patel**: Data curation; formal analysis; funding acquisition; investigation; methodology; project administration; resources; software; supervision; validation; visualization. **Gurpreet Panesar**: Data curation; formal analysis; investigation; methodology; project administration; resources; software; supervision; validation; visualization. **Kunal Soni**: Data curation; formal analysis; investigation; methodology; project administration; resources; software; supervision; validation; visualization. **Kartik B. Dhami**: Data curation; formal analysis; investigation; methodology; project administration; resources; software; supervision; validation; visualization. **Nirja Patel**: Data curation; formal analysis; investigation; methodology; project administration; resources; software; supervision; validation; visualization. **Sohilkhan R. Pathan**: Data curation; formal analysis; investigation; methodology; project administration; resources; software; supervision; validation; visualization. **Hardil P. Majmudar**: Data curation; formal analysis; investigation; methodology; project administration; resources; software; supervision; validation; visualization.

## CONFLICT OF INTEREST

The authors declare no conflict of interest.

## TRANSPARENCY STATEMENT

The lead author Vishal V. Bhende affirms that this manuscript is an honest, accurate, and transparent account of the study being reported; that no important aspects of the study have been omitted; and that any discrepancies from the study as planned (and, if relevant, registered) have been explained.

## Data Availability

The data that support the findings of this study are openly available in Authorea at https://doi.org/10.22541/au.164156721.15230264/v1.
